# Conspiratorial Thinking During COVID-19: The Roles of Paranoia, Delusion-Proneness, and Intolerance of Uncertainty

**DOI:** 10.3389/fpsyt.2021.698147

**Published:** 2021-08-18

**Authors:** Emmett M. Larsen, Kayla R. Donaldson, Megan Liew, Aprajita Mohanty

**Affiliations:** Neuroscience of Emotion, Cognition, and Psychopathology Laboratory, Department of Psychology, Stony Brook University, Stony Brook, NY, United States

**Keywords:** conspiracy, conspiracy theory, schizotypy, anxiety, individual differences, epistemic

## Abstract

The COVID-19 global pandemic has left many feeling a sense of profound uncertainty about their world, safety, and livelihood. Sources espousing misinformation and conspiracy theories frequently offer information that can help make sense of this uncertainty. Individuals high in intolerance of uncertainty (IU) may be particularly impacted by the impoverished epistemic environment and may thus be more drawn to conspiratorial thinking (CT). In the present work, we show across 2 studies (*N* = 519) that COVID-19-specific CT is associated with higher levels of IU as well as delusion-proneness, and paranoia. Furthermore, delusion-proneness and paranoia explained the relationship between IU and CT and emerged as independent partial correlates of CT even when controlling for other facets of schizotypy. In contrast, anxiety did not explain the relationship between IU and CT. Overall, our findings highlight the importance of individual differences in IU, delusion-proneness and paranoia in the development of CT in the context of the acute uncertainty of a global crisis, in which conspiracy theories are more prevalent and salient. Informational intervention designs may benefit from leveraging the body of work demonstrating the efficacy of targeting IU to incite meaningful changes in thinking.

## Introduction

The COVID-19 global pandemic has caused many to feel a sense of profound uncertainty about their world, safety, and livelihood. States of uncertainty are inherently aversive (1) and may provoke quick, less deliberative decisions. In the context of *epistemic* uncertainty–wherein the true state of the world is unclear and must be inferred–decision-makers must adjudicate between competing options. This epistemic uncertainty leaves decision-makers vulnerable to the influence of external sources which may vary in their degrees of veracity. Conspiracy theories are often present in this “marketplace” of frameworks for making sense of an uncertain world, and one key psychological appeal of conspiracy theories may be their promise of providing certainty (2). Perhaps unsurprisingly, conspiracy theories specifically regarding COVID-19 began to spread in the 1st weeks of the outbreak, proliferated quickly soon after (3), and have remained stably prevalent (4). The willingness to endorse conspiracy theories–or *conspiratorial thinking* (CT)–bears a causal link to diminished acceptance of science and decreased engagement in prosocial behaviors (5). Preliminary work has further shown that COVID-19-specific CT is associated with decreased engagement in recommended health behaviors (4, 6, 7). Importantly, the young adult demographic may be at greatest risk of being swayed by COVID-19 misinformation (8), less likely to adhere to health guidelines (9), and more likely to be asymptomatic spreaders of the virus (10). An important task of behavioral scientists in a global health crisis is to identify individual traits and predispositions that are associated with CT or susceptibility to misinformation in young adults in order to inform interventions and methods of science communication (11, 12).

CT arises frequently in times of crisis (13). However, individual differences leading to CT at these times are unclear, as most empirical research examining CT has been conducted outside the context of global crisis, making the COVID-19 pandemic a uniquely important circumstance in which to study these effects. Furthermore, the “epistemic motive” thought to drive CT may be particularly salient when available information is impoverished (2), such as during the COVID-19 pandemic. This affords a unique opportunity to examine these processes as they occur naturally, with COVID-19-related conspiracy theories being both prevalent and salient throughout the pandemic.

Intolerance of uncertainty (IU) is an individual difference in the degree to which uncertainty is experienced as aversive (14). Given the role of CT in resolving uncertainty, those high in IU may be more willing to accept conspiratorial explanations [e.g., (15)] due to their promise of explanatory power. Preliminary empirical work suggests that IU is indeed associated with CT (16). Additionally, experimental manipulation of uncertainty salience alters the way in which evidence for and against conspiracy theories is evaluated (17). The naturally-occurring state of heightened uncertainty due to the COVID-19 pandemic may produce an analogous modulation. So far, no study has directly tested the relationship between IU and COVID-19 CT, though evidence has begun to emerge for a relationship between IU and *generic* CT during the pandemic (18, 19). Furthermore, younger adults not only endorse greater COVID-19-related CT (8, 20) but also report higher levels of IU during the COVID-19 pandemic (21), underscoring the importance of examining their relationship in this demographic.

Another set of cognitive traits and predispositions that have been shown to constitute susceptibility to development of CT (22–24) involve the need to favor/defend bizarre or elaborate beliefs in the face of contradictory evidence. Such a predisposition is also associated with *delusion-proneness* (25), which some have hypothesized functions as a protectant against undesired possible conclusions (26–28). Similarly, one hypothesized function of CT is to protect cherished beliefs by discrediting contradictory evidence as the product of a conspiracy (2, 29). Indeed, empirical studies consistently show relationships between delusion-proneness and CT (30–32). Delusion-proneness is one dimension in a cluster of traits known as *schizotypy*, which also includes proneness to unusual perceptual experience and social and experiential deficits [e.g., (33)]. Paranoia–a construct highly related to schizotypy (34) characterized by mistrust and beliefs that others harbor malintentions—is also associated with CT (23, 30, 35), including in the context of COVID-19-specific CT (36), and this relationship may in fact be separable from that between delusion-proneness and CT (23, 30, 35). Some evidence suggests that schizotypal traits are higher in younger adults (37), indicating the importance of examining whether these individual differences contribute to the higher prevalence of COVID-19-related CT's in this demographic.

Finally, delusion-proneness and IU may bear a relationship to one another. For example, The high conviction characteristic of delusion-like beliefs may serve to *minimize uncertainty* regarding future states (26), and findings in clinical samples support this, showing that severity of delusional thinking is associated with greater intolerance of uncertainty (38, 39). Given the possibly shared epistemic function of both CT and delusional thinking for reducing uncertainty and the hypothesized relationship of IU and delusion-proneness, the relationship between IU and CT may be mediated by delusion-proneness. It is also possible that the relationship of IU with CT operates via heightened affective distress (anxiety). For instance, van Prooijen and Jostmann (17) demonstrate that higher levels of distress following an uncertainty manipulation increased CT. In addition, although it is thought that CT typically fails to assuage anxiety created by unmet epistemic needs (2), preliminary work has suggested a “protective” relationship between CT and anxiety/depression during the COVID-19 pandemic (40). Finally, it has long been thought that IU directly causes and maintains anxious distress itself (14). Greater IU is associated with increased anxiety [e.g., (41)], and IU is considered a transdiagnostic target in anxiety treatment (42). Taken together, these relationships suggest that anxiety is a possible intermediary factor that explains the relationship of IU with CT.

Overall, theoretical and empirical work suggests a particular constellation of individual differences that predispose to CT. The present work seeks to characterize the interplay amongst individual differences in explaining COVID-19 CT and evaluate possible mediators in two samples of young adults. In Study 1 we aim to examine the hypothesized relationships between IU, delusion-proneness, paranoia, and CT. In Study 2 we assess whether these relationships replicate while also increasing statistical power to conduct mediation analysis across the two samples. We hypothesized that IU, anxiety, delusion-proneness, and paranoia would be positively associated with COVID-19 CT. Finally, we examined whether the relationship between IU and CT can be explained by schizotypy-related dimensions (delusion-proneness and paranoia) and/or by affective dimensions like anxiety, both of which are related to IU and CT.

## Study 1

### Method

Data were collected electronically from 261 participants between the dates of April 21st, 2020 and May 8th, 2020. Twenty-one individuals (8%) who spent <10 min completing surveys were excluded from analyses due to suspected inattentiveness, leaving 240 participants in the final sample. Median completion time for the remainder of the sample was 27.3 min. All study participants were Stony Brook University (SBU) undergraduates who received course credit for participation. The study measures were collected in the context of a larger study on attitudes during COVID-19 [see (43) for other variables assessed in the Study 1 sample]. Full participant demographics are presented in Table 1. Informed consent was obtained prior to study participation, and study procedures were approved by the SBU Institutional Review Board (IRB).

**Table 1:** Demographics and questionnaire descriptives for Study 1.

	**N (%)**	**Mean (SD)**	**α**
Age	–	19.88 (2.39)	–
Gender			
Female	150 (62.5)	–	–
Male	90 (37.5)	–	–
Race/Ethnicity			
White/Caucasian	73 (30.4)	–	–
Hispanic/Latino	37 (15.4)	–	–
Black	18 (7.5)	–	–
Asian/Pacific Islander	101 (42.1)	–	–
Other	11 (4.6)	–	–
Measure			
COVID-19 CT	–	34.62 (12.19)	0.93
COVID-19 CT # Items	–	2.35 (2.94)	0.84
IUS	–	31.51 (11.03)	0.94
GPTS	–	19.33 (14.57)	0.94
MIS	–	3.28 (3.20)	0.81
PAS	–	1.31 (2.55)	0.88
Negative Schizotypy	–	5.61 (4.22)	0.78

*COVID-19 CT, Conspiratorial thinking related to COVID-19; COVID-19 CT # Items, number of CT items endorsed as either “Probably True (4)” or “Definitely True (5).” STAIS, State Anxiety; IUS, Intolerance of Uncertainty Scale; GPTS, Green et al. Paranoid Thought Scales; MIS, Magical Ideation Scale; PAS, Perceptual Aberration Scale*.

#### Individual Difference Measures

Questionnaires were administered via Qualtrics survey software. Demographic questions were first, followed by remaining questionnaires in randomized order.

##### Intolerance of Uncertainty

IU was measured using the short form Intolerance of Uncertainty Scale [IUS; (14, 44)]. The IUS assesses “excessive tendency of an individual to consider it unacceptable that a negative event may occur, however small the probability of its occurrence” (13, p. 932). It has been validated in young adult samples, in which it demonstrates very high internal consistency (44). The 12 short form items were summed for analysis (45).

##### Schizotypy

Schizotypal traits were assessed using the short Wisconsin Schizotypy Scales (33). Each scale contains 15 yes/no items with the sum of items comprising the scale score. These scales include two measures of “positive schizotypy,” assessing hallucination-proneness [Perceptual Aberration Scale (PAS)] and delusion-proneness [Magical Ideation Scale (MIS)], as well as two measures of “negative schizotypy” assessing physical and social anhedonia. The negative subscales were combined for the present analyses for use as a single index. The short-form Wisconsin scales have been well-validated in young adults and demonstrate even higher internal consistency than their long-form counterparts (33).

##### Paranoia

Paranoia was assessed using the Revised Green et al. Paranoid Thought Scale [GPTS; (46, 47)]. The GPTS contains two sub-scales of 10 and 8 items, yielding measures of ideas of social reference and persecution, respectively, over the past month. The Revised GPTS has excellent psychometric characteristics compared to its long-form counterpart and has been validated for diverse clinical and non-clinical sample-types (46). Because the present work does not hypothesize differential relationships with the two domains of paranoia, the two subscales were summed to form a single paranoia index utilized in analyses, which has been done in past work as well (48). Main analyses are also reproduced using the two subscales independently in Supplementary Tables 3, 4.

#### Conspiratorial Thinking

COVID-19 CT was evaluated using 16 items drawn from online news outlets reporting on common conspiracy theories (see Supplementary Table 1). Response options and instructions were adapted from the Generic Conspiracist Beliefs Scale (49), a broad measure of engagement with conspiracy theories. The score used in analyses represents mean endorsement across all items. In addition, we calculated the number of items rated as either *Probably true* or *Definitely true* for each participant to descriptively characterize overall CT.

#### Data Availability

Data and syntax used for analyses in both Study 1 and Study 2 are available at https://osf.io/dtzne/.

#### Analyses

All analyses used SPSS 27. Bivariate Pearson correlations assessed the relationships between endorsement of COVID-19 CT and individual difference measures; spearman correlations were used in place of these for variables that demonstrated skewness values > 1.0. Linear regressions with CT as the outcome assessed specificity of schizotypy domains when controlling for shared variance. As a robustness check, linear regressions were repeated using log-transformed versions of variables in cases where significant skew was observed. Data visualizations were created using the ggplot2 package (50) of the R programming environment (51).

### Results

#### Descriptive Characteristics and Correlations

**Figure 1:**
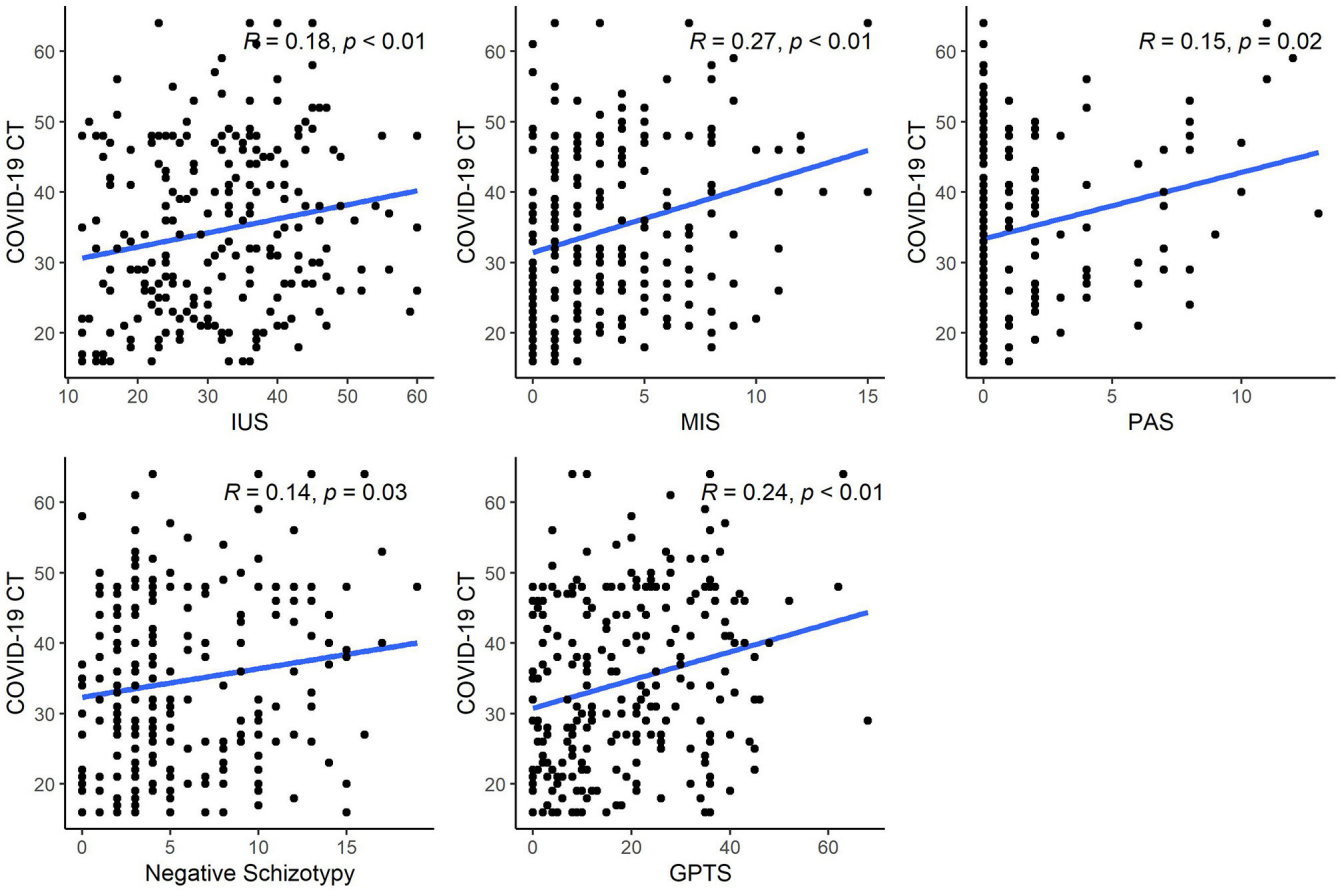
Correlation coefficients for MIS and PAS represent Spearman's Rho while all others represent Pearson's R. CT, Conspiratorial thinking; IUS, Intolerance of Uncertainty Scale; MIS, Magical Ideation Scale; PAS, Perceptual Aberration Scale; GPTS, Green et al. Paranoid Thought Scale.

Descriptive statistics for self-report measures are presented in Table 1 (interrelationships between scales are presented in Supplementary Table 2). Skewness values >1.0: were observed for MIS (1.19) and PAS (2.43); thus Spearman correlations were computed in lieu of Pearson correlations for these variables. Correlation analyses revealed significant associations (*p* < 0.05) of COVID-19 CT with all variables (Figure 1). These relationships remained significant after applying FDR correction for multiple comparisons.

#### Specificity of Schizotypy Dimensions Associated With CT

A linear regression analysis with COVID-19 CT as outcome assessed the hypothesized relationships with MIS and GPTS when controlling for variance shared between them and with PAS and negative schizotypy. Significant independent coefficients emerged for both MIS (β = 0.173, *p* = 0.024) and GPTS (β = 0.148, *p* = 0.034); no other partial coefficients accounted for significant variance (*p*s > 0.30). In total, the adjusted *R*^2^ for this model was 0.079 [*F*_(4,232)_ = 6.09, *p* < 0.001]. In addition, when conducting this analysis using log-transformed versions of MIS and PAS, we observed the same results: significant coefficients emerged for MIS (β = 0.218, *p* = 0.003) and GPTS (β = 0.147, *p* = 0.038), all other *p*s > 0.2.

### Discussion

Study 1 found preliminary support for the hypotheses that individual differences in IU and schizotypy (which may be specific to delusion-proneness and paranoia) contribute to COVID-19-related CT. Given the rapidly-changing nature of both the COVID-19 pandemic as well as the availability of information to the public, we deemed it important to reexamine these relationships at a second time point within the pandemic when atmospheric uncertainty may be different. In addition, pooling data across Studies 1 and 2 provided statistical power to examine possible mediators of the relationship between IU and CT (52). Thus, Study 2 aimed to accomplish both the goals of testing replicability and obtaining power to test mediation.

## Study 2

### Method

Data were collected electronically from 258 participants between the dates of October 15th, 2020 and November 19th, 2020. Sixteen individuals (6%) who spent <10 min completing study surveys were excluded from analyses due to suspected inattentiveness, leaving 242 participants in the final sample. Median completion time for the remainder of the sample was 38.1 min. Missing data was handled by retaining participants for each analysis who answered all items for the relevant questionnaire(s). All study participants were SBU undergraduates who received course credit for participation. Participant demographics are presented in Table 2. Informed consent was obtained prior to study participation, and study procedures were approved by the SBU IRB.

**Table 2:** Demographics and questionnaire descriptives for Study 2.

	**N (%)**	**Mean (SD)**	**α**
Age	–	19.26 (1.53)	–
Gender			
Female	158 (65.3)	–	–
Male	84 (34.7)	–	–
Race/ethnicity			
White/Caucasian	57 (23.6)	–	–
Hispanic/Latino	29 (12.0)	–	–
Black	13 (5.4)	–	–
Asian/Pacific Islander	136 (56.2)	–	–
Other	7 (2.9)	–	–
Measure			
COVID-19 CT	–	33.76 (11.37)	0.92
COVID-19 CT # items	–	1.95 (2.31)	0.76
STAIS	–	44.85 (12.23)	0.95
IUS	–	35.71 (9.13)	0.90
GPTS	–	21.18 (15.30)	0.94
MIS	–	3.86 (3.40)	0.81
PAS	–	1.90 (3.25)	0.90
Negative schizotypy	–	5.50 (4.38)	0.80

*COVID-19 CT, Conspiratorial thinking related to COVID-19; COVID-19 CT # Items, number of CT items endorsed as either “Probably True (4)” or “Definitely True (5).” STAIS, State Anxiety; IUS, Intolerance of Uncertainty Scale; GPTS, Green et al. Paranoid Though Scales; MIS, Magical Ideation Scale; PAS, Perceptual Aberration Scale*.

#### Measures

Administration procedures as well as measures of IU, schizotypy, paranoia, and CT in Study 2 were identical to those in Study 1. In Study 2 we additionally assessed anxiety using the State-Trait Anxiety Inventory-State scale [STAIS; (53)], which contains 20 items measuring momentary experience of apprehension, tension, nervousness, and worry.

#### Analyses

Primary analyses were conducted identically to those in Study 1. In addition, we conducted mediation analysis using the PROCESS macro version 3.5 (54) to examine whether the relationship between IU and CT was explained by anxiety, delusion-proneness, or paranoia. Significance in these analyses at alpha = 0.05 is indicated by an indirect effect confidence interval that does not include zero.

To obtain appropriate statistical power for mediation analyses, we combined samples from studies 1 and 2. Additionally, to ensure that results were not affected by potential differences between the two study samples, we reran significant models with the inclusion of sample source (Study 1 or Study 2) as a covariate.

### Results

#### Descriptive Characteristics and Correlations

**Figure 2:**
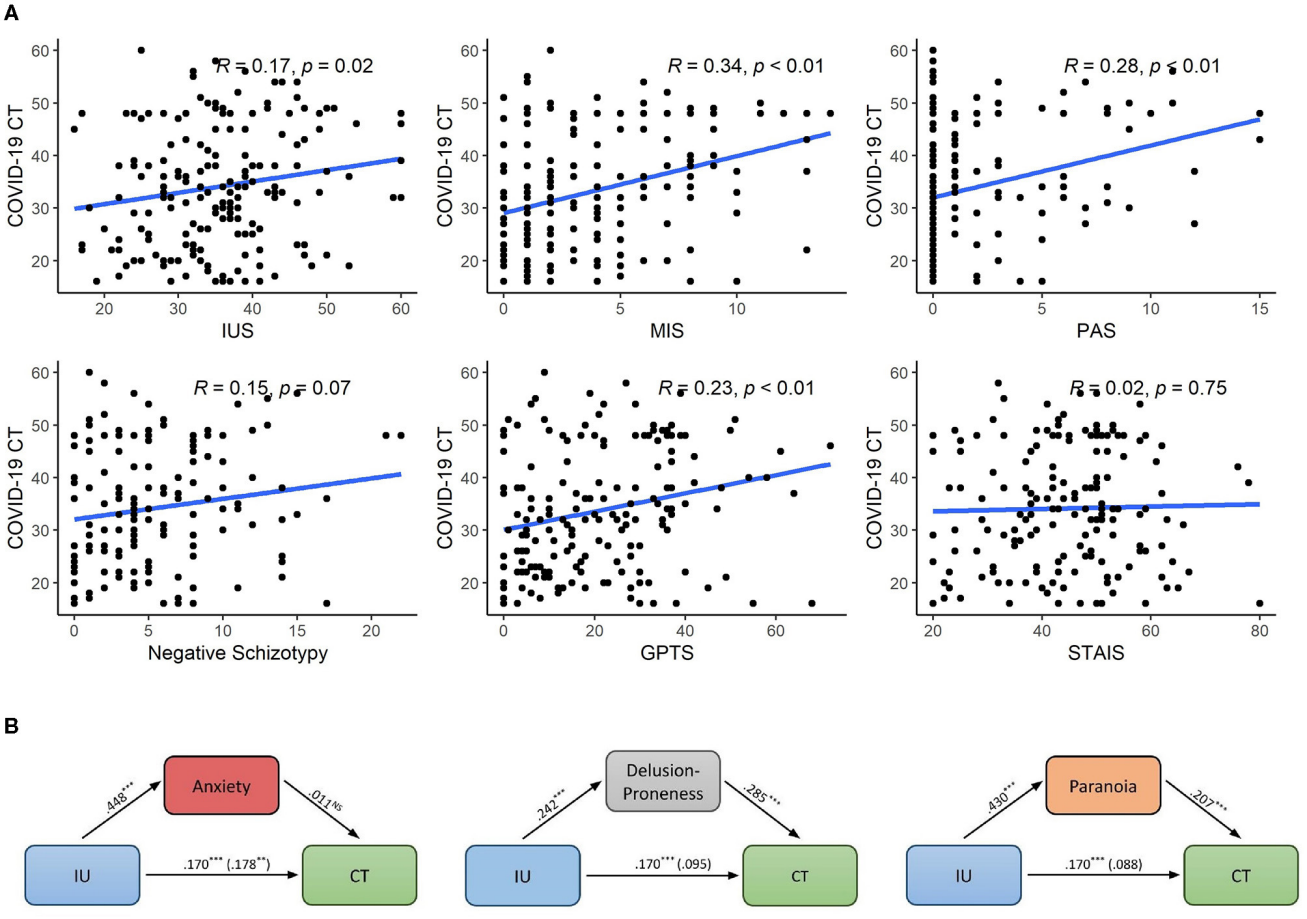
**(A)** Relationships of individual difference measures with CT in Study 2. Correlation coefficients for PAS and Negative schizotypy represent Spearman's Rho while all others represent Pearson's R. **(B)** Mediation paths for three possible factors explaining the relationship between IU and CT. CT, Conspiratorial thinking; IUS, Intolerance of Uncertainty Scale; MIS, Magical Ideation Scale; PAS, Perceptual Aberration Scale; GPTS, Green et al. Paranoid Thought Scale. **p < 0.01, ***p < 0.001.

Descriptive statistics for self-report measures are presented in Table 2 (interrelationships between scales are presented in Supplementary Table 2). Skewness values >1.0: were observed for PAS (1.91) and Negative schizotypy (1.11); thus Spearman correlations were computed in lieu of Pearson correlations for these variables. Correlation analyses revealed significant associations of COVID-19 CT with all variables (*p* < 0.05) except anxiety and negative schizotypy, thus replicating 4 of the 5 relationships identified in Study 1 (Figure 2A). These relationships remained significant after applying FDR correction.

#### Specificity of Schizotypy With CT

A linear regression analysis with COVID-19 CT as outcome assessed the hypothesized relationships with MIS and GPTS when controlling for variance shared with PAS and negative schizotypy. As in Study 1, a significant independent coefficient emerged for MIS (β = 0.350, *p* = 0.013). Contrary to Study 1, we did not observe an independent effect of GPTS (β = −0.005, *p* = 0.965). No other partial coefficients accounted for significant variance (*p*s > 0.30). The adjusted *R*^2^ for this model was 0.081 [*F*_(4,80)_ = 2.845, *p* = 0.029]. In addition, when conducting this analysis using log-transformed versions of negative schizotypy and PAS, we observed the same results: significant coefficients emerged for MIS only (β = 0.333, *p* = 0.017), all other *p*s > 0.5.

#### Mediators of IU to CT

Mediational analyses examined whether relationships between IU and CT could be accounted for by anxiety (STAIS), delusion-proneness (MIS), or paranoia (GPTS). We first entered STAIS as the mediating variable and found a non-significant indirect effect (β = 0.005, SE = 0.027, 95% CI = −0.047, 0.060). With MIS as the mediating variable, we observed a significant indirect effect (β = 0.069, SE = 0.019, 95% CI = 0.035, 0.110). Likewise, with paranoia as the mediating variable, we observed a significant indirect effect (β = 0.089, SE = 0.026, 95% CI = 0.041, 0.142). Figure 2B depicts these pathways. To assess directionality of the two significant models, we entered IUS as the mediating variable, and did not observe a significant indirect effect for either the model from MIS or from GPTS to CT (95% CIs both included zero). Lastly, we reran the two significant models when controlling for source sample (Study 1 or Study 2), revealing that both models retained significant indirect effects of MIS and GPTS respectively.

### Discussion

Results from Study 2 replicated those of Study 1 with two exceptions: a non-significant correlation of negative schizotypy with CT as well as slight difference in the result of the linear regression, such that delusion-proneness was the only significant factor associated with CT among schizotypy-related variables (whereas Study 1 found that paranoia was also significant). This divergent finding may point to a different relationship between paranoia and COVID-19-related CT in early 2020 as opposed to late 2020. Lastly, Study 2 provided evidence that the relationship between IU and CT is explained by delusion-proneness and paranoia, but not by anxiety.

## General Discussion

Conspiracy theories have had heightened salience and prevalence during the COVID-19 pandemic (3, 8, 36). Ambient uncertainty has also been high due to constantly changing availability of information, and it is hypothesized that such uncertainty may activate the epistemic needs associated with CT (2). This work sought to shed light on the individual differences leading to increased COVID-19-CT amongst young adults, a demographic at heightened risk for CT (8, 20). Study 1 examined the hypothesized relationships between IU, delusion-proneness, paranoia, and CT; Study 2 assessed whether these relationships replicated and examined possible mediators of the IU-CT relationship. Study 1 showed that higher IU and schizotypy were related to increased COVID-19-CT. Examination of specific components of schizotypy showed that delusion-proneness and paranoia independently related to CT while other related facets did not. Study 2 replicated these findings (with one slight difference in the specificity analysis) and additionally showed that delusion-proneness and paranoia, but not anxiety, were significant mediators of the association between IU and CT.

Earlier theoretical [e.g., (15)] and empirical (16) work has identified the relationship between IU and CT in line with the present findings. It has also been strongly suggested that CT is most pronounced in historical times of crisis (13); our study provides evidence for the relationship of IU and CT in such a time of heightened uncertainty. While some preliminary work during the pandemic has shown an association between IU and generic CT (18, 19), the present findings are the first to establish the relationship of IU with COVID-19-specific CT. Some past work outside the context of a global crisis has suggested the relationship between IU and CT is less than clear (16), and it is possible that our sample exhibited an amplified relationship between these factors due to the particular salience of environmental uncertainty and uncontrollability caused by the COVID-19 pandemic. Indeed, the *salience* of uncertainty is thought to affect the relationship of IU to CT (17), and experimental evidence suggests that CT can be both increased and decreased by manipulating sense of control of outcomes (55).

Our findings are also in line with past work demonstrating relationships between schizotypy and CT (22–24) and extend findings to context-specific CT in a major public health crisis. Furthermore, while past work has not often examined independent facets of schizotypy, here we show specificity in relationships with schizotypal symptoms. Neither negative schizotypy nor the perceptual aberration component of positive schizotypy were independently related to CT; rather, effects were specific to delusion-proneness and (in Study 1 only) paranoia. Past studies that have examined relationships with particular symptom/trait domains are consistent with the notion that delusion-proneness and paranoia may have unique contributions to CT (23, 30, 35). Our findings suggest that the independent effect of paranoia may be less stable and influenced by pandemic-related factors, as it did not replicate in the late-2020 sample. The effect of delusion-proneness is also consistent with work showing that CT relates to a tendency to perceive non-existent agency and intentionality (56, 57), an effect that is stronger in individuals with schizotypal traits (57). Future work should investigate the degree to which hypersensitive agency detection may explain the relationship between delusion-proneness and CT.

The finding that delusion-proneness and paranoia mediated the IU-CT relationship is a novel empirical contribution to the literature. It is consistent with theoretical work on cognitive functions played by CT and delusion-like thinking (2, 26, 27) as well as empirical evidence of the relationship between delusions and IU in psychosis (38, 39). However, no previous study to our knowledge has assessed dynamics of these variables with relation to CT. Furthermore, the mediation effect sizes were substantial: delusion-proneness and paranoia explained 40.6 and 52.4%, respectively, of the relationship between IU and CT. Our results provide preliminary evidence that for individuals experiencing IU and with proneness to delusion-like thinking or mistrust, conspiracies represent an appealing option for belief. Conversely, our finding that anxiety did *not* explain the relationship between IU and CT provides an important contrast and clue about the psychological dynamics that result in CT in times of uncertainty. That is, while those who experience uncertainty as particularly aversive also tend to experience higher levels of anxiety and distress (particularly in the context of the global pandemic), this distress may not be the mechanism by which conspiracy theories come to be an appealing epistemic anecdote for uncertainty. Rather, our results suggest that prior tendencies for delusion-proneness and paranoid ideation are more likely to drive those experiencing aversive uncertainty to settle on conspiratorial conclusions. Overall, this constitutes evidence for processes involved in reasoning and belief formation as opposed to emotional responses to uncertain threats in explaining the relation between IU and CT in young adults, which may have implications for intervention.

Our cross-sectional design is a limitation of the present work. While we posit that the personality constructs evaluated were pre-existing and constitute vulnerability factors for CT, such an inference can only be truly supported via longitudinal measurements. Additionally, little work has assessed measurement invariance in the time of COVID-19, and it is possible that self-report instruments do not operate identically in a context of environmental uncertainty posed by the pandemic (58). One final limitation of this work is that it took a relatively broad approach to assessing endorsement of CT. There is considerable variation in the degree to which conspiracy theories are divorced from reality, and some types of CT may originate from socio-political, demographic and economic realities (59, 60). It is thus likely that future work would benefit from taking a more nuanced approach to assessment of types of CT and potentially separable factors associated with them. Such examinations would be crucial to quantifying the portions of CT phenomena that are and are not explained by the psychopathology-related factors examined here–a point that is further highlighted by noting the relatively small percent of variance accounted for by the variables we examined here (~8%).

Future work should attempt to identify cognitive mechanisms of the relationships shown here to better understand the nature of traits that predispose to CT during a global health crisis. It remains unclear whether processes such as cognitive insight and analytical thinking, which are diminished in individuals with schizotypal traits and CT proneness (61), play significant roles in heightened threat and uncertainty. Additionally, given the potentially-dissociable relationships of CT with delusion-proneness and paranoia, further work is needed to identify whether they differ in their pathways to CT, cognitive mediators, and behavioral correlates. Such work would also clarify the precise targets for informational interventions to protect vulnerable populations from the allure of conspiracy theories. Importantly, the present conclusions are drawn from a sample of young adults, a population that is both more likely to endorse COVID-19-specific CT and to be asymptomatic spreaders of the COVID-19 virus. Given the predisposition amongst this demographic to both IU and schizotypal traits, the present findings represent highly relevant targets for future interventions. It is possible that additional factors may be contributory to COVID-19-specific CT in other populations–including other populations of young adults with different demographic compositions; future studies should seek to explore potential effects of demographic group on the effectiveness of intervention strategies by recruiting larger and more diverse samples.

Despite the scientific community condemning conspiratorial thinking and misinformation spread early in the COVID-19 pandemic (62, 63), it is likely that more concerted efforts are needed to stymie the proliferation of misinformation that contributes to CT during the remainder of the pandemic and beyond. While the optimal strategy for fighting misinformation is unclear, recent attempts as well as cumulative efforts made by the scientific community to correct misinformation show promise in combating the COVID-19 “infodemic” (64, 65). These findings suggest that targeting cognitive factors (rather than affective responses), such as engaging analytic thinking for COVID-19 related information, will improve intervention strategies since such thinking is less habitual for some with a predilection for CT (61). In addition, efforts might benefit from consulting the growing body of research that has supported the efficacy of targeting IU itself to incite meaningful changes in thinking (66, 67).

## Data Availability Statement

The datasets presented in this study can be found in online repositories. The names of the repository/repositories and accession number(s) can be found at: https://osf.io/dtzne/.

## Ethics Statement

The studies involving human participants were reviewed and approved by Stony Brook University Institutional Review Board. All participants indicated informed consent for study procedures.

## Author Contributions

EL and AM conceived the study and conducted the analyses. EL and ML collected and curated the data. EL, KD, ML, and AM wrote and revised the manuscript. All authors contributed to the article and approved the submitted version.

## Conflict of Interest

The authors declare that the research was conducted in the absence of any commercial or financial relationships that could be construed as a potential conflict of interest.

## Publisher's Note

All claims expressed in this article are solely those of the authors and do not necessarily represent those of their affiliated organizations, or those of the publisher, the editors and the reviewers. Any product that may be evaluated in this article, or claim that may be made by its manufacturer, is not guaranteed or endorsed by the publisher.
