# Supplementary material for: Conspiratorial Thinking During COVID-19: The Roles of Paranoia, Delusion-Proneness, and Intolerance of Uncertainty
Source: Front Psychiatry. 2021 Aug 18;12:698147. doi: 10.3389/fpsyt.2021.698147 (PMC8416269; doi:10.3389/fpsyt.2021.698147)
Supplement: Supplementary file 1 [file Data_Sheet_1.docx]

***Supplementary Material***

**Supplementary Table S1.** COVID-19 Conspiracy Theories

| 1. | The US or UK (or other western government) created or weaponized the coronavirus, intentionally introducing it to the population in an attempt to attack the chinese economy |
| --- | --- |
| 2. | The US or UK (or other western government) created or weaponized the coronavirus, intentionally introducing it to the population in an attempt to make money by selling vaccines. |
| 3. | The Chinese government created or weaponized the coronavirus, intentionally introducing it to the population in an attempt to attack the US/western economy. |
| 4. | The Chinese government created or weaponized the coronavirus, intentionally introducing it to the population in an attempt to make money by selling vaccines. |
| 5. | COVID-19 was engineered by governmental bodies of another country (e.g. Canada), possibly as a bio-weapon, for unknown reasons. |
| 6. | Bill Gates (via the Bill and Melinda Gates Foundation) teamed with a UK-based company (Pirbright Institute) which produces COVID-19 vaccines to create the virus in order to profit from selling the vaccines. |
| 7. | COVID-19 is a result of radiation poisoning from 5G cell phone signals which weaken the immune system. Evidence for this is that Wuhan (the first Chinese city to report COVID-19) was also one of the first Chinese cities to get 5G internet. |
| 8. | COVID-19 originated on a meteorite that exploded in China in 2019, bringing the virus here from space. |
| 9. | COVID-19 was created by scientists by modifying a strand of Human Immunodeficiency Virus (HIV). |
| 10. | COVID-19 resulted from the Chinese diet containing bats and other animals which are known to carry dangerous pathogens |
| 11. | The situation with COVID-19 has been intentionally instigated by people with interests in removing Donald Trump from office in the upcoming election (e.g. the Democratic party). |
| 12. | The situation with COVID-19 has been intentionally instigated by Donald Trump and members of the Republican party in order to increase the likelihood of his being reelected in the upcoming election. |
| 13. | The Chinese President, Xi Jinping, and his political agenda has been a key cause of the spread of the COVID-19. |
| 14. | There is a vaccine or cure for COVID-19 that the government is currently withholding from citizens. |
| 15. | COVID-19 was created as a method of intentional “population control”. |
| 16. | Governments have deliberately reported much lower numbers of infections and deaths caused by COVID-19 in order to keep the true numbers from the public. |

*Note.* Questionnaire instructions: “There has been debate about various important issues related to COVID-19. This brief survey is designed to assess your beliefs about some of these subjects. Please indicate the degree to which you believe each statement is likely to be true on the following scale: Definitely not true; Probably not true; Not sure/cannot decide; Probably true; Definitely true.”

**Supplementary Table S2.** Correlations amongst scales

| **STUDY 1** |  |  |  |  |  |  |  |
| --- | --- | --- | --- | --- | --- | --- | --- |
| **Measure** | ***1*** | ***2*** | ***3*** | ***4*** | ***5*** | ***6*** | ***7*** |
| 1. COVID-CT | -- | .180^**^ | .107 | .239*^***^* | .198*^**^* | .254*^**^* | .140*^*^* |
| 2. IUS |  | -- | .495*^***^* | .445*^**^* | .212*^**^* | .360*^**^* | .185*^**^* |
| 3. STAIS |  |  | -- | .325*^**^* | .243*^**^* | .187*^**^* | .249*^***^* |
| 4. GPTS |  |  |  | -- | .351*^**^* | .372*^**^* | .253*^**^* |
| 5. PAS |  |  |  |  | -- | .533*^**^* | .308*^**^* |
| 6. MIS |  |  |  |  |  | -- | .206*^**^* |
| 7.Negative |  |  |  |  |  |  | -- |
| **STUDY 2** |  |  |  |  |  |  |  |
| **Measure** | ***1*** | ***2*** | ***3*** | ***4*** | ***5*** | ***6*** | ***7*** |
| 1. COVID-CT | -- | .172^*^ | .028 | .235^**^ | .288^***^ | .337^***^ | .156 |
| 2. IUS |  | -- | .444^***^ | .384^***^ | .075 | .056 | .222^**^ |
| 3. STAIS |  |  | -- | .453^***^ | .110 | .053 | .196^*^ |
| 4. GPTS |  |  |  | -- | .223^**^ | .344^***^ | .210^**^ |
| 5. PAS |  |  |  |  | -- | .527^***^ | .453^***^ |
| 6. MIS |  |  |  |  |  | -- | .172^*^ |
| 7.Negative |  |  |  |  |  |  | -- |

*Note.* **p*<.05, ***p*<.01, ****p*<.001. Abbreviations: CT = conspiratorial thinking. IUS = Intolerance of Uncertainty Scale. STAIS = State Trait Anxiety Inventory - State scale. GPTS = Greet et al Paranoid Though Scales. MIS = Magical Ideation Scale. PAS = Perceptual Aberration Scale. Negative = Negative Schizotypy.

**Supplementary Table S3.** Correlations of GPTS subscales with COVID-19 CT

| **STUDY 1** |  |  |  |
| --- | --- | --- | --- |
| **Measure** | ***1*** | ***2*** | ***3*** |
| 1. COVID-CT | -- | .160^*^ | .260^***^ |
| 2. GPTS-A |  | -- | .645*^***^* |
| 3. GPTS-B |  |  | -- |
| **STUDY 2** |  |  |  |
| **Measure** | ***1*** | ***2*** | ***3*** |
| 1. COVID-CT | -- | .205^**^ | .230^**^ |
| 2. GPTS-A |  | -- | .716*^***^* |
| 3. GPTS-B |  |  | -- |

*Note.* **p*<.05, ***p*<.01, ****p*<.001. Abbreviations: CT = conspiratorial thinking. GPTS = Greet et al Paranoid Though Scales.

**Supplementary Table S4.** Regression of COVID-19 CT on schizotypy scales

| ***DV=COVID-19 CT*** | **Study 1** | | **Study 2** | |
| --- | --- | --- | --- | --- |
|  | **Adjusted R^2^=.081** | | **Adjusted R^2^=.078** | |
|  | **β** | ***p*** | **β** | ***p*** |
| **MIS** | .17 | .03 | .37 | .01 |
| **PAS** | .02 | .77 | -.04 | .76 |
| **GPTS-A** | -.01 | .86 | .08 | .61 |
| **GPTS-B** | .18 | .04 | -.09 | .59 |
| **Negative** | .05 | .45 | .13 | .30 |

*Note*. Abbreviations: CT = conspiratorial thinking. GPTS = Greet et al Paranoid Though Scales. MIS = Magical Ideation Scale. PAS = Perceptual Aberration Scale. Negative = Negative Schizotypy.
